# Constructing an Asymmetric Covalent Triazine Framework to Boost the Efficiency and Selectivity of Visible‐Light‐Driven CO_2_ Photoreduction

**DOI:** 10.1002/advs.202402645

**Published:** 2024-05-13

**Authors:** Guang‐Dong Qi, Dan Ba, Yu‐Jie Zhang, Xue‐Qing Jiang, Zihao Chen, Miao‐Miao Yang, Jia‐Min Cao, Wen‐Wen Dong, Jun Zhao, Dong‐Sheng Li, Qichun Zhang

**Affiliations:** ^1^ College of Materials and Chemical Engineering Key Laboratory of Inorganic Nonmetallic Crystalline and Energy Conversion Materials China Three Gorges University Yichang Hubei 443002 P. R. China; ^2^ Hubei Three Gorges Laboratory Yichang Hubei 443007 P. R. China; ^3^ Department of Materials Science and Engineering Department of Chemistry Center of Super‐Diamond and Advanced Films (COSDAF) & Hong Kong Institute of Clean Energy City University of Hong Kong Hong Kong SAR 999077 P. R. China

**Keywords:** asymmetric covalent triazine framework, efficient selectivity, photocatalytic CO2 reduction

## Abstract

The photocatalytic reduction of CO_2_ represents an environmentally friendly and sustainable approach for generating valuable chemicals. In this study, a thiophene‐modified highly conjugated asymmetric covalent triazine framework (As‐CTF‐S) is developed for this purpose. Significantly, single‐component intramolecular energy transfer can enhance the photogenerated charge separation, leading to the efficient conversion of CO_2_ to CO during photocatalysis. As a result, without the need for additional photosensitizers or organic sacrificial agents, As‐CTF‐S demonstrates the highest photocatalytic ability of 353.2 µmol g^−1^ and achieves a selectivity of ≈99.95% within a 4 h period under visible light irradiation. This study provides molecular insights into the rational control of charge transfer pathways for high‐efficiency CO_2_ photoreduction using single‐component organic semiconductor catalysts.

## Graphical abstract

A thiophene‐modified highly conjugated asymmetric covalent triazine framework (As‐CTF‐S) has been constructed. As‐CTF‐S exhibits excellent the photogenerated charge separation, leading to the efficient conversion of CO_2_ to CO during photocatalysis. This study provides a promising path for D–A type CTFs as highly effective heterogeneous catalysts for the selective photoreduction of CO_2_ under gaseous phase with no toxic sacrificial agent.

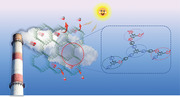

## 1 Introduction

In recent years, fossil fuels have served as the primary energy source to meet the demands of global economic and social development. However, the excessive use of fossil fuels has led to significant carbon dioxide emissions, a poor carbon cycle, and serious environmental and climate issues.^[^ ^1^, ^2^, ^3^, ^4^ ^]^ Consequently, there is an urgent need to develop effective strategies or technologies to reduce atmospheric carbon dioxide levels and rebalance the carbon cycle. The photocatalytic reduction of carbon dioxide has emerged as a promising solution.^[^ ^5^, ^6^ ^]^ Since the first report of CO_2_ photoreduction catalyzed by TiO_2_ in 1979, there have been significant efforts to develop various inorganic semiconductor photocatalysts capable of converting CO_2_ into fuel.^[^ ^7^, ^8^, ^9^, ^10^ ^]^ Researchers have explored transition metal complexes,^[^ ^11^ ^]^ inorganic or hybrid semiconductors,^[^ ^12^, ^13^ ^]^ and metal‐modified zeolites.^[^ ^14^ ^]^ A notable example includes binary graphite carbon nitride (g‐C_3_N_4_).^[^ ^15^, ^16^ ^]^ However, the inefficiency and the poor selectivity in reducing and converting inert CO_2_ into CO remain challenges. Therefore, developing more effective and highly selective photocatalysts for CO_2_ conversion is highly desirable. Metal‐free catalysts exhibit enhanced stability due to their covalent bonding, and they are environmentally benign and cost‐effective. Carrier separation commonly impedes photocatalyst efficiency. By redesigning molecular structures, we can mitigate this issue, potentially establishing it as a superior photocatalyst.^[^ ^17^, ^18^ ^]^

As a novel type of non‐metallic materials, covalent organic frameworks,^[^ ^19^ ^]^ especially covalent triazine frameworks (CTFs), have garnered significant attention in catalytic supports,^[^ ^20^ ^]^ gas capture and storage,^[^ ^21^, ^22^, ^23^ ^]^ energy conversion,^[^ ^24^ ^]^ and catalysis.^[^ ^25^, ^26^ ^]^ However, in photocatalytic reduction, the presence of simple conjugated structures leads to rapid recombination of photoexcited carriers, thereby hindering the generation of excitons and subsequently reducing both selectivity and product yield. To overcome this challenge, researchers are focusing on constructing D–A systems to improve CTFs performance. Wang et al. devised a photosensitizing system incorporating electron D–A units using heptathiophene‐based cucurbituril, thereby enhancing photocatalytic activity through enhanced separation and transfer of photoexcited carriers.^[^ ^27^ ^]^ Likewise, Fan et al. documented benzodithiophene‐based covalent triazine framework materials (BDT‐CTFs), employing donor–acceptor motifs to adjust photocatalytic activity.^[^ ^28^ ^]^ Tan et al. demonstrated that N‐doped fluorene (carbazole), possessing the strongest electron‐donating ability, achieved the highest photocatalytic performance among POPs. This highest performance can be attributed to the enhanced charge transfer efficiency and reduced charge recombination within these heteroatom‐doped donor–acceptor CTFs.^[^ ^29^ ^]^ Jin et al. presented a photocatalytic electron transfer system, fabricating D–A1–A2 conjugated polymers for photocatalytic hydrogen evolution.^[^ ^30^ ^]^ Zhang et al. developed asymmetric covalent triazine frameworks as highly efficient single‐component semiconductor photocatalysts for organic transformations. The integration of four distinct molecular donor–acceptor modules enables intramolecular energy transfer cascades, leading to exceptional photocatalytic performance in organic transformations.^[^ ^25^ ^]^ Nonetheless, significant advancements have been made in the prompt recombination of photoexcited carriers in D–A type covalent triazine frameworks. However, the utilization of single‐component intramolecular energy transfer in D–A CTFs for carbon dioxide reduction remains unexplored.

**Figure 1:**
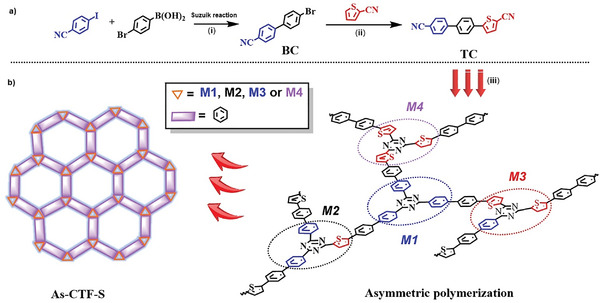
Synthesis routes of the As‐CTF‐S: a) The synthesis of two monomers. b) Polymerization reaction of monomer.

Thus, a thiophene‐modified highly‐conjugated asymmetric covalent triazine framework (As‐CTF‐S) was developed for this purpose. In our approach (**Figure** **1**), 4‐iodobenzonitrile and 4‐bromobenzeneboronic acid were coupled to synthesize 4‐bromo‐4‐cyanobenzene. This intermediate was further coupled with 2‐cyanothiophene to create As‐CTF‐S. Under visible light exposure without photosensitizers or organic sacrificial agents, As‐CTF‐S showed exceptional photo‐catalytic activity, achieving a rate of 353.2 µmol g^−1^ and a selectivity of ≈99.95% within 4 h. This study highlights the significance of asymmetry in enhancing charge separation efficiency in D–A CTFs and offers a strategic guideline for designing efficient photocatalysts.

## 2 Results and Discussion

**Figure 2:**
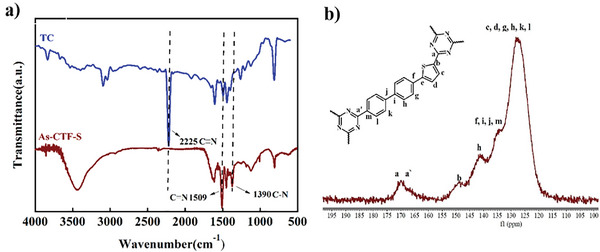
a) FTIR spectra of As‐CTF‐S and monomer. b) Solid state ^13^C CPNMR spectra of As‐CTF‐S.

The CTF was synthesized through a polymerization reaction using 5‐(4′‐cyano [1,1′‐biphenyl]−4‐yl) thiophen‐2‐cyano, named As‐CTF‐S (Detailed synthesis methods are described in the Experimental Section). The FT‐IR spectra of the synthesized CTFs (shown in **Figure** **2**a) revealed the disappearance of the C≡N stretching vibration at 2225 cm^−1^ from the monomers. Simultaneously, new stretching vibration bands for C═N at 1509 cm^−1^ and C─N at 1390 cm^−1^ emerged, indicating the successful formation of triazine units. These spectroscopic findings suggest that the poly‐condensation of monomers led to the creation of the triazine framework structures.^[^ ^31^ ^]^ Additionally, the identification of triazine units within CTFs was further supported by a chemical shift of 170 ppm observed in the solid‐state ^13^C‐CPNMR spectrum (Figure 2b), corresponding to the triazine carbon. The other chemical shifts were assigned to the respective carbons in As‐CTF‐S.

The morphology of As‐CTF‐S was observed through SEM characterization, revealing sub‐micron dimensions (Figure S1, Supporting Information). This feature is thought to stem from the relatively low degree of cross‐linking in As‐CTF‐S. Thermogravimetric analysis (TGA), as shown in Figure S2 (Supporting Information), demonstrates that As‐CTF‐S maintains thermal stability up to ≈300 °C. The N_2_ adsorption–desorption isotherms were conducted at 77 K. The isotherms of the As‐CTF‐S follow a complex type IV trend, exhibiting a steep increase at low *P*/*P*_0_ values, followed by a continuous and monotonic increase showing an H3‐type hysteresis loop. This complex trend exhibits a certain amount of micropores and mesopores and an external surface. As shown in Figure S3 (Supporting Information), the specific Brunauer–Emmett–Teller (BET) surface area of As‐CTF‐S is 17 m^2^ g^−1^. The NLDFT pore‐size distribution profiles indicate that As‐CTF‐S possessed a micropore size of 2.0 nm and mesopore peaks centered at 16.0 nm. In addition, the CO_2_ adsorption capacity of As‐CTF‐S was evaluated at 298 K. As shown in Figure S4 (Supporting Information), CO_2_ uptake capacity for As‐CTF‐S reaches 8 cm^3^ g^−1^. The relatively low adsorption capacity may be due to the amorphous nature of the material. Powder X‐ray diffraction (PXRD) patterns reveal the amorphous nature of As‐CTF‐S, marked by the lack of distinct crystalline peaks, as shown in Figure S5 (Supporting Information). This amorphous characteristic is corroborated by the typical diffraction patterns and is a contributing factor to its relatively low BET surface area.

**Figure 3:**
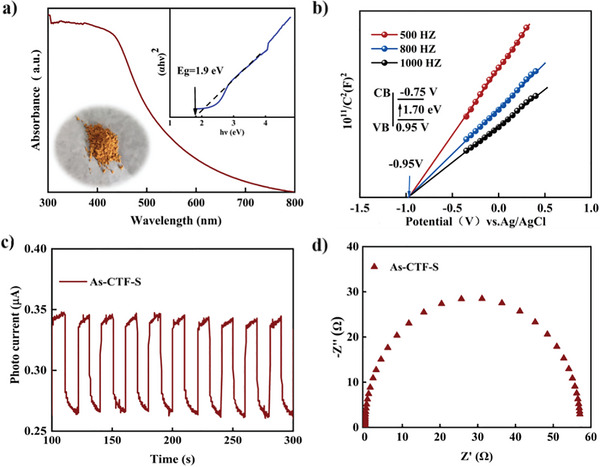
a) UV–vis spectra of As‐CTF‐S. b) Mott‐Schottky plots of As‐CTF‐S in 0.2 M Na_2_SO_4_ aqueous solution. (Inset: the energy diagram of the CB and VB levels of As‐CTF‐S). c) Photocurrent transient response of As‐CTF‐S. d) Electrochemical impedance spectroscopies of As‐CTF‐S.

The UV–vis diffuse reflectance spectra (DRS) were utilized to assess the absorption properties of As‐CTF‐S. **Figure** **3**a shows that As‐CTF‐S captures a broad range of visible light, particularly in the 400–600 nm area. The optical bandgap (*E*_g_) of As‐CTF‐S was determined using the Kubelka−Munk (K–M) method, as depicted in the inset of Figure 3a, revealing an *E*_g_ of 1.9 eV. This relatively narrow bandgap enables more efficient photo‐induced electron transitions. We conducted an in‐depth analysis of the conduction band (CB) and valence band (VB) positions through Mott−Schottky plot measurements at 500, 800, and 1000 Hz frequencies, as shown in Figure 3b. These measurements determined that the CB of As‐CTF‐S is situated at −0.75 V versus NHE, while the VB of As‐CTF‐S lies at +1.15 V. This positioning indicates that the photogenerated electrons in As‐CTF‐S are suitable for the reduction of CO_2_ to CO −0.53 V vs NHE), and the VB of As‐CTF‐S is adequately positive for oxidizing H_2_O to O_2_ (+ 0.82 V vs NHE). The efficiency of charge separation in As‐CTF‐S is demonstrated by their photoelectric properties, where the photo‐current indicates the movement of generated charges under intermittent visible light exposure. As shown in Figure 3c, As‐CTF‐S produces a significant photo‐current upon light exposure. Electrochemical impedance spectroscopy (EIS) analysis, presented in Figure 3d, was used to evaluate the electrical conductivity of CTFs. The Nyquist plot for As‐CTF‐S exhibits a diameter of only 60 Ω, indicating a high capacity for charge migration within the CTFs, which is advantageous for the photo‐catalytic process.

**Figure 4:**
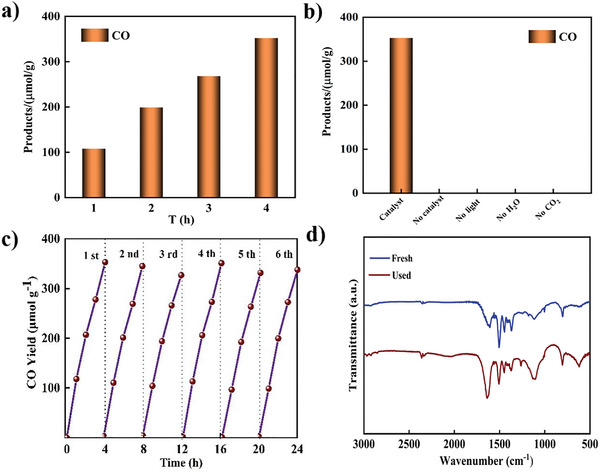
a) Time‐course plots of products with As‐CTF‐S as the photocatalyst. b) Control experiments of CO_2_ photoreduction. c) Recycling performance of As‐CTF‐S. d) The FTIR spectra of As‐CTF‐S before and after the photoreduction.

The CO_2_ photo‐reduction activity was conducted under visible light irradiation (λ > 420 nm) at room temperature using the Labsolar‐6A All glass automatic online trace gas analysis System from Beijing PerfectLight. We employed the prepared sample as the photocatalyst in a gas‐solid reaction mode with water vapor, without the use of sacrificial agents, organic solvents, or photosensitizers. Gas chromatography was utilized to identify the main reduction products. As‐CTF‐S demonstrated superior photocatalytic performance, achieving the highest activity of 353.2 µmol g^−1^ and a selectivity of ≈99.95% within a 4 h period (**Figure** **4**a). The exceptional photocatalytic activity and selectivity of As‐CTF‐S can be attributed to the incorporation of the thiophene unit into the triazine polymerization network. This modification broadens the light absorption spectrum of the catalyst and promotes an asymmetric structure, enhancing photo‐induced electron separation and transfer. To further investigate the mechanism behind photo‐induced CO_2_ reduction, control experiments were conducted using As‐CTF‐S as the photocatalyst. In these experiments, the absence of CO_2_, the catalyst, water vapor, and light led to no detectable products (Figure 4b), underscoring that As‐CTF‐S effectively facilitates CO_2_ conversion under mild conditions with high selectivity for converting gaseous CO_2_ to CO. The produced CO was confirmed to result from CO_2_ reduction rather than from the catalyst or reaction equipment. In addition, the photocatalytic cycling stability was evaluated by cyclic experiments. After six continuous cycles, the conversion yield for CO was still maintained up 95%, suggesting the good stability of As‐CTF‐S. Furthermore, to investigate the long‐term stability of the catalyst's performance, we carried out a continuous catalytic test for 40 h (Figure S13, Supporting Information). The results indicated that the catalytic performance remained stable without significant decay. The results indicated that the catalytic performance remained relatively stable, with no significant decay detected. FT‐IR spectra analysis before and after the reaction revealed no significant changes, indicating that As‐CTF‐S maintains a stable chemical structure within the conjugate network (Figure 4d). Furthermore, As‐CTF‐S was compared with similar chemical structure CTF photocatalysts for CO_2_ reduction, as outlined in Table S1 (Supporting Information). As‐CTF‐S not only surpasses similar CTF photocatalysts in CO_2_ reduction in terms of both performance and selectivity but also outperforms most comparable catalysts currently reported in the literature.

**Figure 5:**
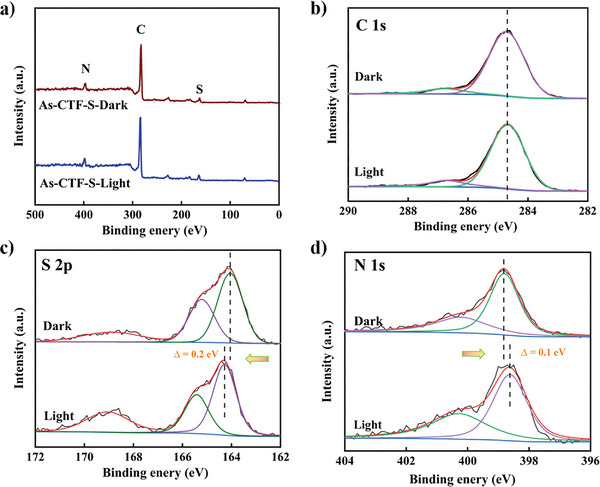
a) The XPS survey spectra of As‐CTF‐S, b) High‐resolution C 1s XPS spectrum. c) High‐resolution S 2p XPS spectrum. and d) High‐resolution N1s XPS spectrum.

X‐ray Photoelectron Spectroscopy (XPS) results (**Figures** **5**a,b) provided insight into the XPS survey spectrum for As‐CTF‐S, revealing the presence of C, N, and S elements. The S 2p spectrum of As‐CTF‐S showed binding energies at 163.4 and 164.4 eV, corresponding to the S 2p3/2 and S 2p1/2 states of sulfur within the thiophene units (Figure 5c). The high‐resolution N1s spectra of these polymers exhibited a binding energy of 397.7 eV, indicating the presence of triazine rings (C═N─C) in CTFs (Figure 5d). To delve deeper into the photoelectron transfer process in As‐CTF‐S, XPS analyses under illumination were performed to observe changes in the binding energies of different elements with and without light exposure. An observed increase in binding energy under light suggests a reduction in electron cloud density around specific atoms or groups, indicative of electron transfer. Notably, the binding energy of the S 2p peak increased under visible light, while the N1s peak's binding energy decreased, suggesting electron donation by sulfur atoms and acceptance by nitrogen atoms due to this structural effect.^[^ ^25^, ^31^, ^32^ ^]^

**Figure 6:**
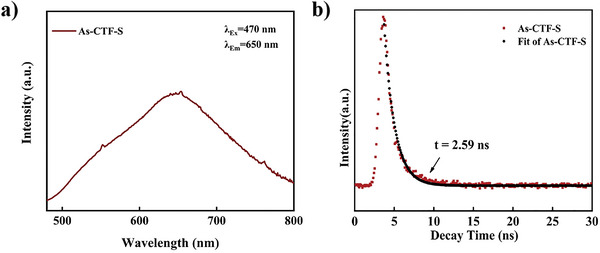
a) The photoluminescence (PL) emission of As‐CTF‐S excited at 470 nm. b) Time‐resolved PL spectra of As‐CTF‐S.

Photoluminescence (PL) spectra, with an excitation wavelength of 470 nm, were used to monitor electronic recombination. The results indicated weak fluorescence emission, suggesting minimal photo‐electron recombination (**Figure** **6**a). Time‐resolved fluorescence spectra at steady‐state emission peaks yielded an average irradiation lifetime of 2.59 ns (Figure 6b), implying that an extended fluorescence lifetime is beneficial for photocatalytic CO_2_ reduction.

**Figure 7:**
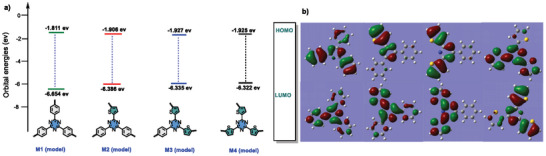
a) The frontier orbital distribution of model molecules. b) The HOMO/LUMO values calculated at the B3LYP/6‐31G (d, p) level.

The photo‐oxidation and reduction capabilities of CTF are significantly affected by its electronic structure. In a donor–acceptor (D–A) system, the electron‐donating unit influences the highest occupied molecular orbital (HOMO) energy level, while the acceptor impacts the lowest unoccupied molecular orbital (LUMO) energy level. Density functional theory (DFT) analysis showed that M4 has the highest HOMO level, while M1 has the highest LUMO level. A decrease in HOMO levels was observed in other D–A models as the presence of the thiophene ring decreased, making M4 the most potential electron donor (**Figures** **7**a,b). Furthermore, calculations revealed that M4 has the narrowest HOMO‐LUMO energy gap (*E*_g_), indicating that electron transitions within it are more readily facilitated. Kinetic competition experiments with methylbenzonitrile and thiophene‐2‐carbonitrile in a 1:1 ratio led to the formation of four D–A model combinations. These experiments ranked the likelihood of forming these combinations as M’2 > M’3 > M’1 > M’4. Combining insights from the literature, theoretical analyses, and experimental results, it is clear that M’2 demonstrates a substantial driving force for the photocatalytic reduction of CO_2_, promoting quick charge separation within the material (Figure S6, Supporting Information).

**Figure 8:**
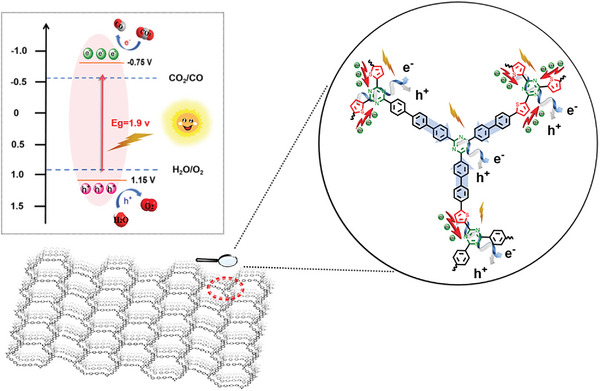
The proposed mechanism of photocatalytic CO_2_ reduction for As‐CTF‐S.

The asymmetric structure, which incorporates four donor–acceptor (D–A) sub‐units, promotes the efficient separation and transfer of charges. This mechanism ensures that the photogenerated electrons are directed toward the triazine ring. Due to its inherent electronic structure that absorbs well, the triazine ring facilitates the conversion of CO_2_ to CO through electron transfer at the active site of a nitrogen atom.^[^ ^33^ ^]^ Additionally, the nitrogen atom within the triazine ring interacts with polarizable CO_2_ molecules via dipole‐quadrupole interactions,^[^ ^34^ ^]^ effectively capturing and concentrating negative electrons. The photocatalytic reduction of CO_2_ to CO occurs in two primary stages. First, the abundant holes on the thiophene and benzene rings oxidize H_2_O to O_2_, producing protons (H_2_O → 1/2 O_2_ + 2H^+^ + 2e^−^). Then, the generated protons combine with CO_2,_ and the photo‐generated electrons collected at the active site of the nitrogen atom aid in CO formation (CO_2_ + 2H⁺ + 2e⁻ → CO + H_2_O)^[^ ^35^, ^36^ ^]^ (**Figure** **8**). Because of the amorphous porous As‐CTF‐S, the photocatalytic reaction may take place in both internal pore space and external surfaces.

## 3 Conclusion

In summary, As‐CTF‐S has emerged as an innovative photocatalyst, designed specifically for the reduction of CO_2_ to CO under visible light exposure. Impressively, As‐CTF‐S delivers exceptional photo‐catalytic performance, achieving a production rate of 353.2 µmol g^−1^ within a 4 h timeframe and showcasing a remarkable selectivity of ≈99.95%. This high level of efficiency is attained without reliance on photosensitizers or organic sacrificial agents. The development of a single‐component system utilizes donor–acceptor (D–A) models to enhance the combination of energy levels and improve charge separation efficiency. This study provides a promising path for the creation of new D–A type CTFs as highly effective heterogeneous catalysts for the selective photoreduction of CO_2_ under the gaseous phase with no toxic sacrificial agent. Further investigation of novel D–A CTFs with various donor–acceptor configurations for photocatalytic application is in progress.

## 4 Experimental Section

### Materials

All chemicals were commercially available and used without further purification. 4‐Bromobenzonitrile (99%), Trifluoromethanesulfonic acid (99%), 2‐cyanothiophene (95%), tetrakis (triphenylphosphine) palladium (99%), potassium carbonate (K_2_CO_3_) and all other solvents were purchased from Sigma–Aldrich. All chemicals and solvents were used as received unless otherwise specified. All reactions were carried out under a nitrogen atmosphere employing standard Schlenk techniques unless otherwise noted.

### Synthesis of 4‐Bromo‐4‐Cyanobiphenyl (BC)

4‐bromophenylboric acid (0.4 g, 1.99 mmol), 4‐bromobenzonitrile (0.91 g, 3.98 mmol), and potassium carbonate (0.55 g, 3.98 mmol) were placed into a 100 mL double‐necked round‐bottomed flask, containing in a mixture of 1,4‐dioxane (50 mL) and water (10 mL). After tetra (triphenylphosphine) palladium (231 mg, 0.2 mmol) was added, the mixture was refluxed under an argon atmosphere for 24 h. After the reaction, the reaction was cooled to room temperature, and the solvent was removed under vacuum. The residue was diluted with dichloromethane and washed with brine. The crude product was purified by column chromatography to obtain BC as white Solid, ^1^H NMR (400 MHz, Chloroform‐d, Figure S7, Supporting Information) *δ* 7.75 – 7.69 (m, 2H), 7.66 – 7.62 (m, 2H), 7.62 – 7.57 (m, 2H), 7.47 – 7.42 (m, 2H); ^13^C NMR (101 MHz, Chloroform‐d, Figure S8, Supporting Information) *δ* 144.4, 138.0, 132.7, 132.3, 128.8, 127.5, 123.2, 118.8, 111.3.

### Synthesis of 5‐(4′‐cyano[1,1′‐biphenyl]−4‐yl) Thiophen‐2‐Cyano (TC)

Under argon atmosphere, 2‐Cyanothiophene (1.09 g, 10 mmol) and BC (1.29 g, 5 mmol) were reacted at 150 °C for 24 h in a flask containing *N, N* dimethylacetamide (12 mL), potassium acetate (1.47 g, 15 mmol) and Palladium acetate (0.002 g, 0.01 mmol). After the reaction was over, the resulting mixture was cooled to room temperature. The resulting solid was filtered to a given yellow‐green crude solid, which was washed with deionized water 3–6 times to obtain TC (Gray‐green powder, 1.5 g, 88.2%). ^1^H NMR (400 MHz, Chloroform‐*d*, CF_3_COOH, Figure S9, Supporting Information) *δ* 9.06 (s, 1H – CF_3_COOH), 7.79 (s, 2H), 7.76 (d, *J* = 3.3 Hz, 2H), 7.74 (d, *J* = 3.3 Hz, 2H), 7.71 – 7.69 (m, 2H), 7.68 (s, 1H), 7.39 (d, *J* = 4.0 Hz, 1H).; ^13^C NMR (101 MHz, Chloroform‐*d*, CF_3_COOH, Figure S10, Supporting Information) *δ* 152.0, 145.2, 140.1, 139.6, 133.2, 133.0, 132.5, 128.2, 128.1, 127.8, 127.2, 124.0, 110.0, 107.2.; ^19^F NMR (565 MHz, CDCl_3,_ Figure S11, Supporting Information)δ – 75.87 (d, *J* = 8.3 Hz).

### Polymerization Reaction of Monomer

Using TC (1.50 g, 3.5 mmol) as the polymerization monomer and trifluoromethanesulfonic acid as the catalyst, the synthesis of As‐CTF‐S was conducted under a 100 °C temperature and argon atmosphere. After the reaction was completed, cool it to room temperature. Subsequently, wash it with deionized water first, followed by washing with ethanol and ethyl acetate three –six times. The obtained samples should be soaked in a diluted ammonia aqueous solution overnight. The obtained sample was dried at room temperature under vacuum conditions to obtain As‐CTF‐S as the final product (Brown powder, 1.41 g, 94%). Elemental analysis, calculated values (%): C, 75.50; N, 9.78; S, 11.20; experimental values (%): C, 73.98; N, 9.83; S, 11.55.

### Characterization

Powder X‐ray diffraction (XRD) was collected by Rigaku Ultima IV diffractometer (CuKα radiation, λ = 1.5406 Å). Thermogravimetric (TG) curves were performed on a NETZSCH 449C thermal analyzer with a heating rate of 10 °C min^−1^ under air atmosphere. The UV–vis diffuse reflection spectroscopy (DRS) was taken using Shimadzu UV–vis spectrophotometer (2550, Japan). Nitrogen adsorption/desorption isotherms were performed on Microtrac BEL BELSORP‐max. The specific surface areas were evaluated by the Brunauer–Emmett–Teller (BET) method and pore size distributions were tested using the density function theory (DFT) method. X‐ray photoelectron spectroscopic (XPS) was investigated by an ESCALAB 250 with a monochromatic Al Kα X‐ray source. Transient photocurrent measurements, electrochemical impedance spectroscopy (EIS), and Mott‐Schottky analyses were performed using an electrochemical workstation (CHI660E).

### Photoelectrochemical Measurements

The photocatalytic reaction of CO_2_ reduction was performed in a 120 mL reactor cell with 1 atm CO_2_ partial pressure. The reaction temperature was kept at 30 °C by a circulating water bath. During the experiments, 5 mg of the catalyst was added to 1 mL of methanol and was sonicated for 10 min. The solution was dispersed on a 35 mm Petri dish and then dried under a vacuum. The deionized water (1 mL) was added to the bottom of the reactor and the Petri dish was fixed above the water. As a low boiling‐point liquid, methanol can easily generate a film of the sample on the petri dish. Before illumination, the atmosphere in the reactor was exchanged with high‐purity CO_2_ gas several times and then the reactor was tightly closed. A 300 W Xe lamp with a 420 nm cutoff optical filter was used as the light source and irradiated from the front of the reactor to start the CO_2_ reduction reaction. After the reaction, the amount of CO was analyzed and quantified by gas chromatography with a detector of Shimadzu GC‐2014 FID, TCD, with argon as the carrier gas. All photoelectrochemical experiments were performed on an electrochemical workstation (CHI660E) containing a three‐electrode system with a 0.2 m Na_2_SO_4_ aqueous solution as an electrolyte. The samples were coated on fluorine‐doped tin oxide (FTO) glass (1 cm^2^) and were employed as the working electrode and Ag/AgCl and platinum wire as the reference electrode and the counter electrode, respectively.

## Conflict of Interest

The authors declare no conflict of interest.

## Supporting information

Supporting Information

## Data Availability

The data that support the findings of this study are available in the supplementary material of this article.
