# Supplementary material for: Constructing an Asymmetric Covalent Triazine Framework to Boost the Efficiency and Selectivity of Visible‐Light‐Driven CO2 Photoreduction
Source: Adv Sci (Weinh). 2024 May 13;11(28):2402645. doi: 10.1002/advs.202402645 (PMC11267385; doi:10.1002/advs.202402645)
Supplement: Supplementary file 1 — Supporting Information [file ADVS-11-2402645-s001.docx]

**Table of Contents**

**Figure S1.** SEM images of the As-CTF-S.

**Figure S2.** The TGA curve of As-CTF-S.

**Figure S3.** N_2_ adsorption-desorption isotherms and pore width distribution curves (insert) of As-CTF-S.

**Figure S4.** The CO_2_ adsorption isotherm of As-CTF-S.

**Figure S5.** Powder X-ray diffraction (PXRD) patterns of As-CTF-S.

**Figure S6.** Competition experiment and product was monitored by TCL.

**Figure S7.** ^1^H NMR spectrum of BC.

**Figure S8.** ^13^C NMR spectrum of BC.

**Figure S9.** ^1^H NMR spectrum of TC.

**Figure S10.** ^13^C NMR spectrum of TC.

**Figure S11.** ^19^F NMR spectrum of CF_3_COOH.

**Figure S12.** EDX elemental mapping and analysis of As-CTF-S.

**Figure 13.** Time-course plots of products with As-CTF-S as the photocatalyst.

**Table S1.** The reported CTF-based photocatalysts for the CO_2_ photoreduction.

**Figure S1**. SEM images of As-CTF-S.


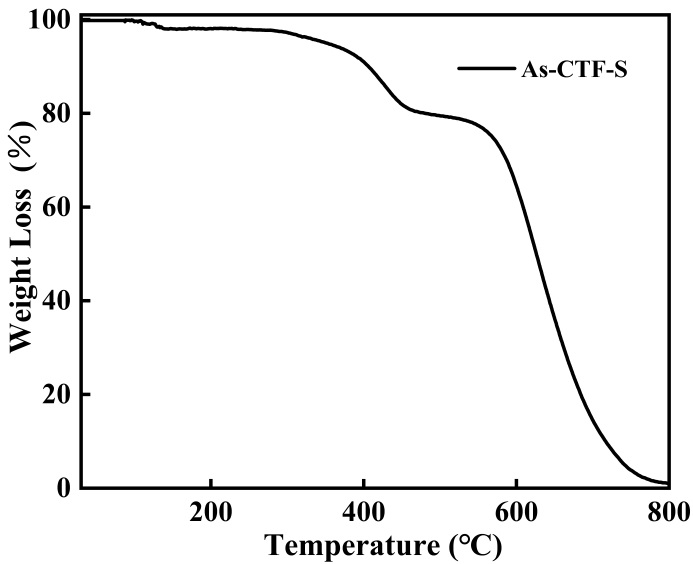


**Figure S2.** The TGA curve of As-CTF-S.


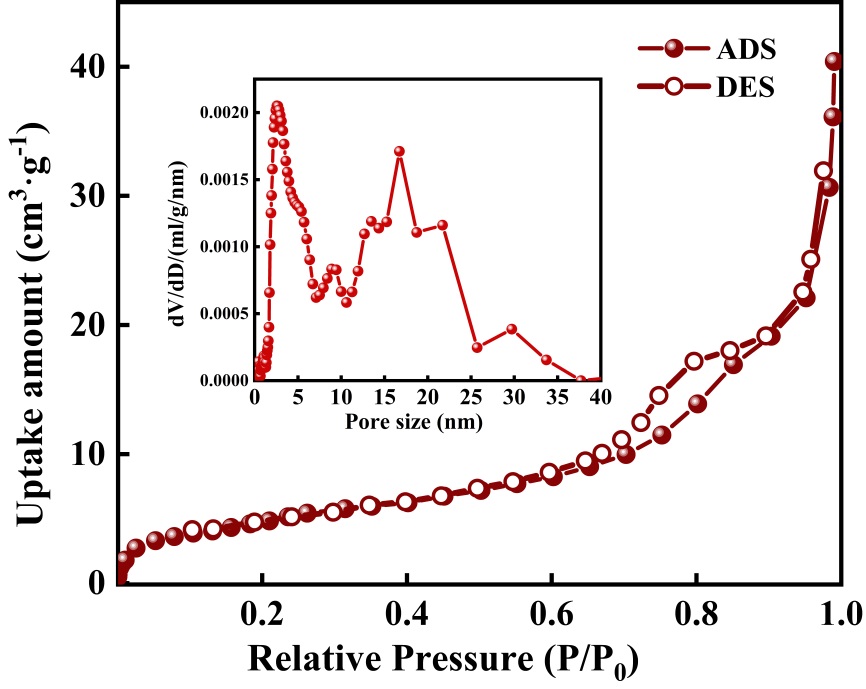


**Figure S3.** N_2_ adsorption-desorption isotherms and pore width distribution curves (insert) of As-CTF-S.


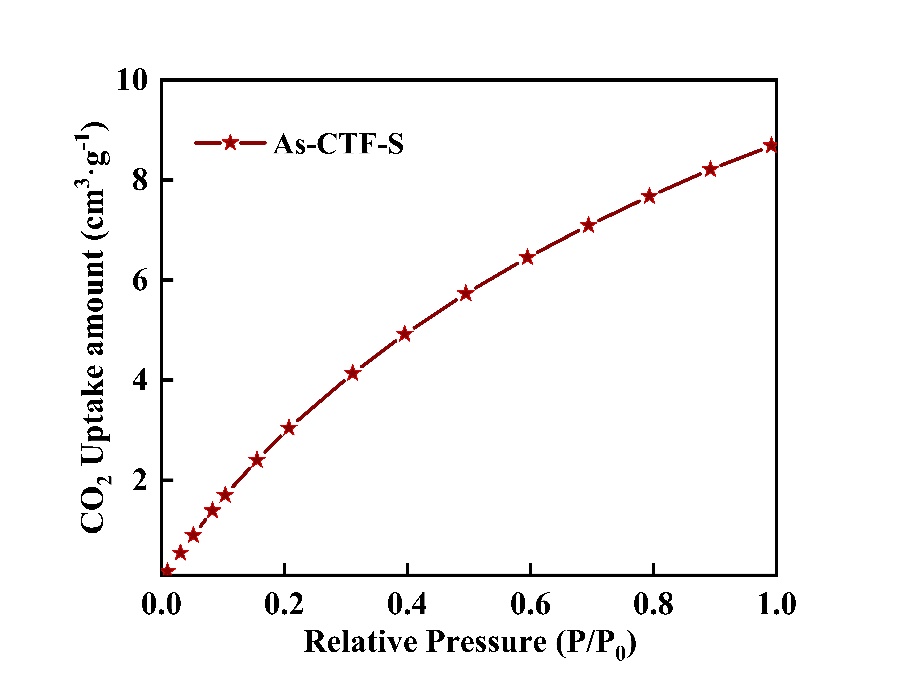


**Figure S4.** The CO_2_ adsorption isotherm of As-CTF-S.


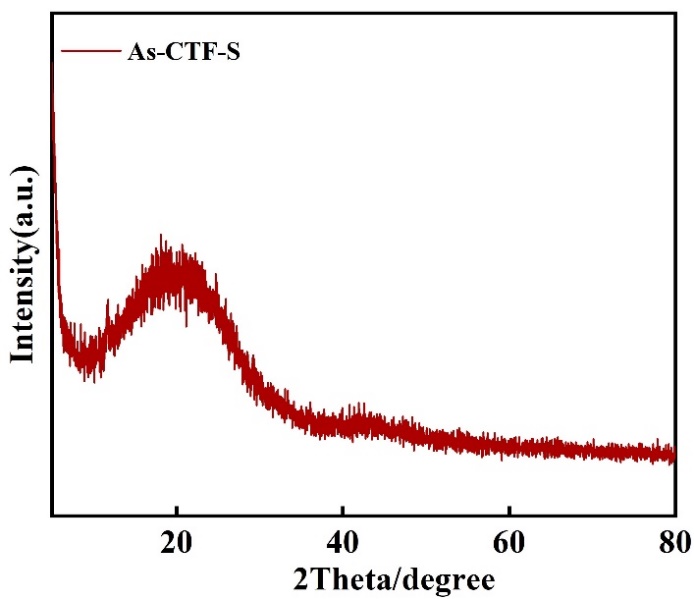


**Figure S5.** Powder X-ray diffraction (PXRD) patterns of As-CTF-S.


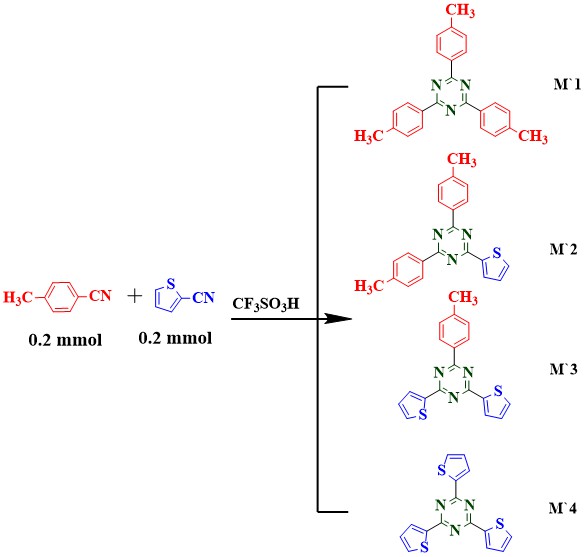

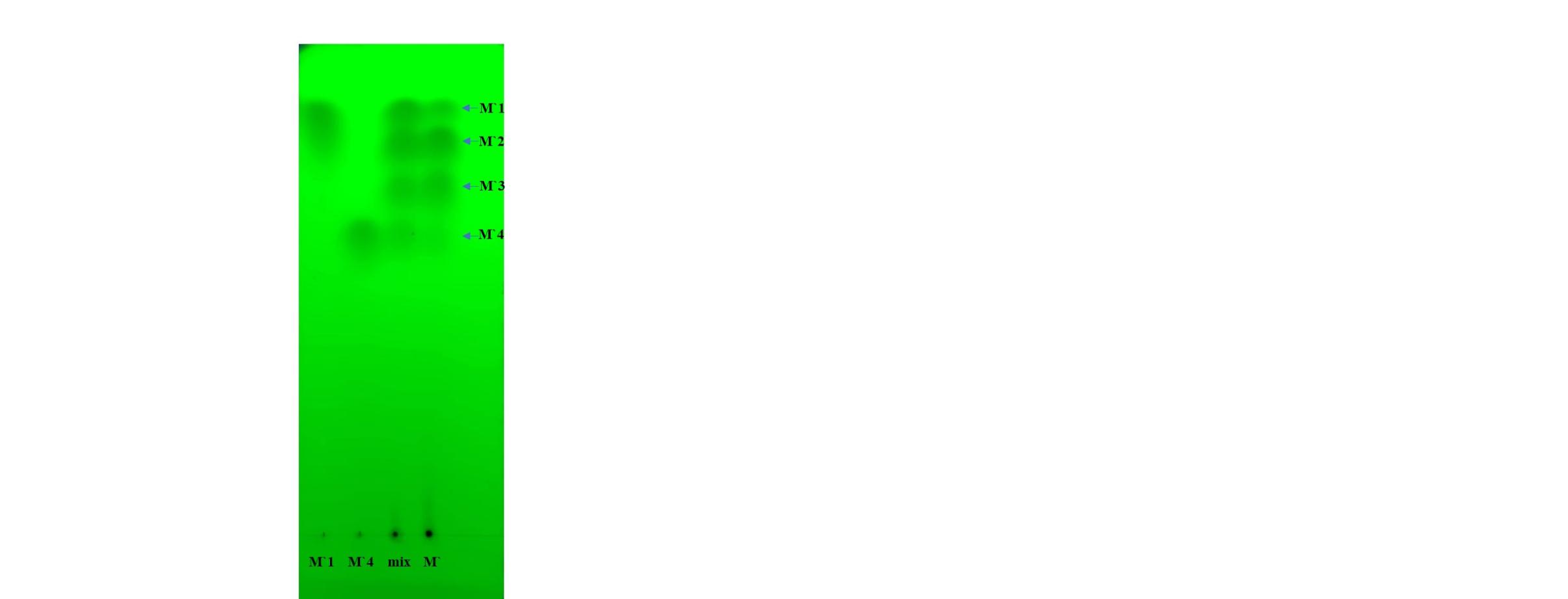


**Figure S6.** Competition experiment and product was monitored by TCL.

A mixture of 4-Methylbenzenecarbonitrile (0.1 mmol) and 2-cyanothiophene (0.1 mmol) was polymerized under similar conditions. The kinetics of the competitive experiment was analyzed using thin-layer chromatography. The results indicated that the probabilities of generating these four modules follow the order of M’2 > M’3 > M’1 > M’4, as determined by TLC plate staining analysis. M`1, white, solid, ^1^H NMR (400 MHz, Chloroform-*d*) δ 9.00 – 8.47 (m, 6H), 7.37 (d, J = 8.0 Hz, 6H), 2.48 (s, 9H). M`4, light brown solid, ^1^H NMR (400 MHz, Chloroform*-d*) δ 8.28 (dd, J = 3.8, 1.3 Hz, 3H), 7.62 (dd, J = 4.9, 1.3 Hz, 3H), 7.21 (dd, J=5.0, 3.7, 3H).


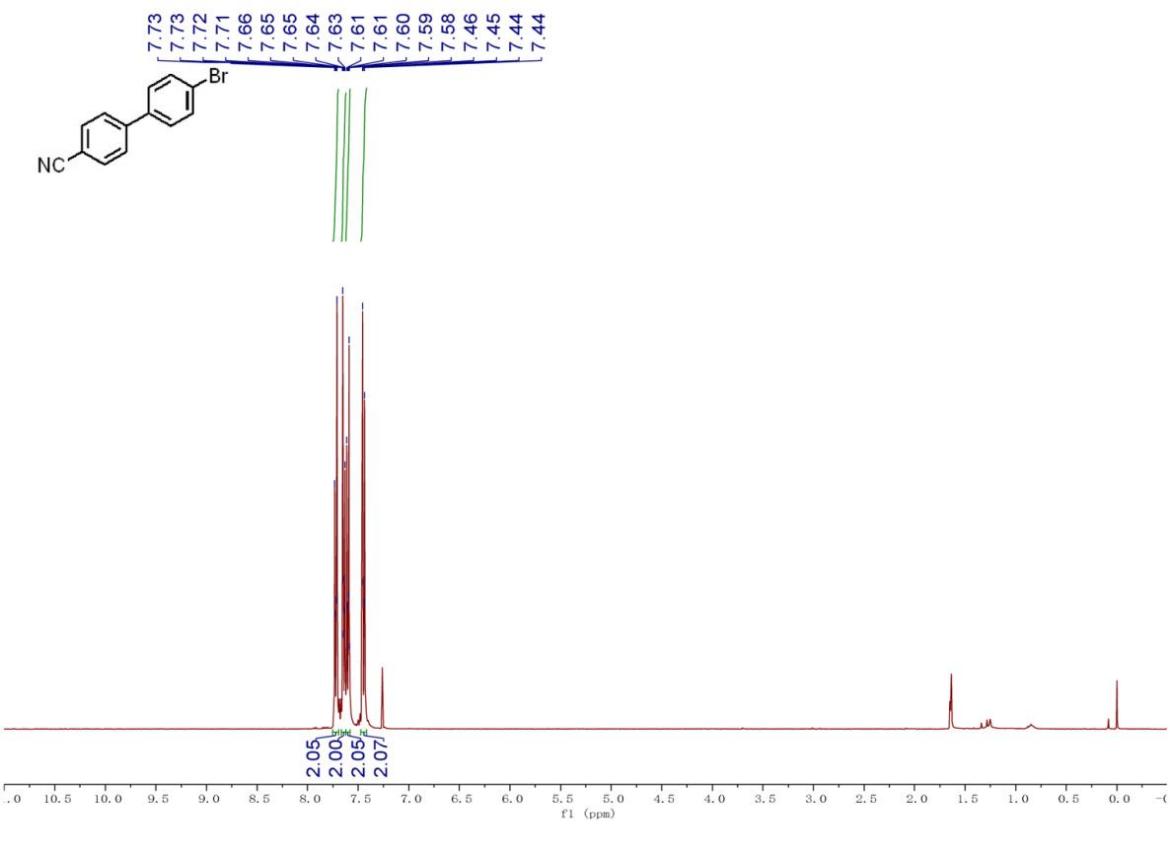


**Figure S7**. ^1^H NMR spectrum of BC.

 **Figure S8**. ^13^C NMR spectrum of BC.


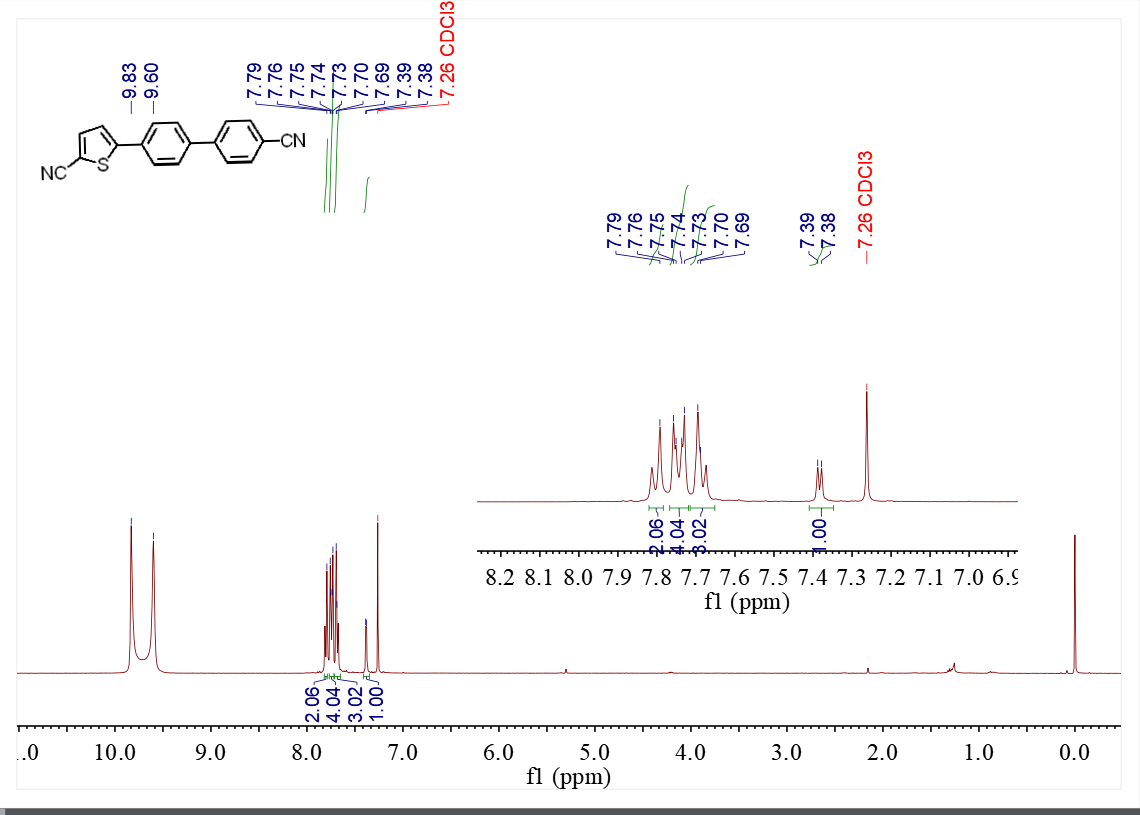


**Figure S9**. ^1^H NMR spectrum of TC.


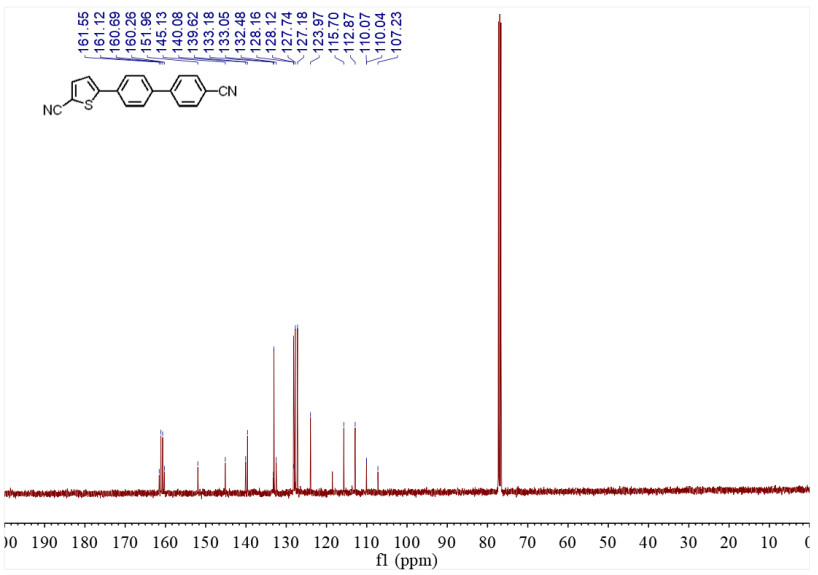


**Figure S10**. ^13^C NMR spectrum of TC.


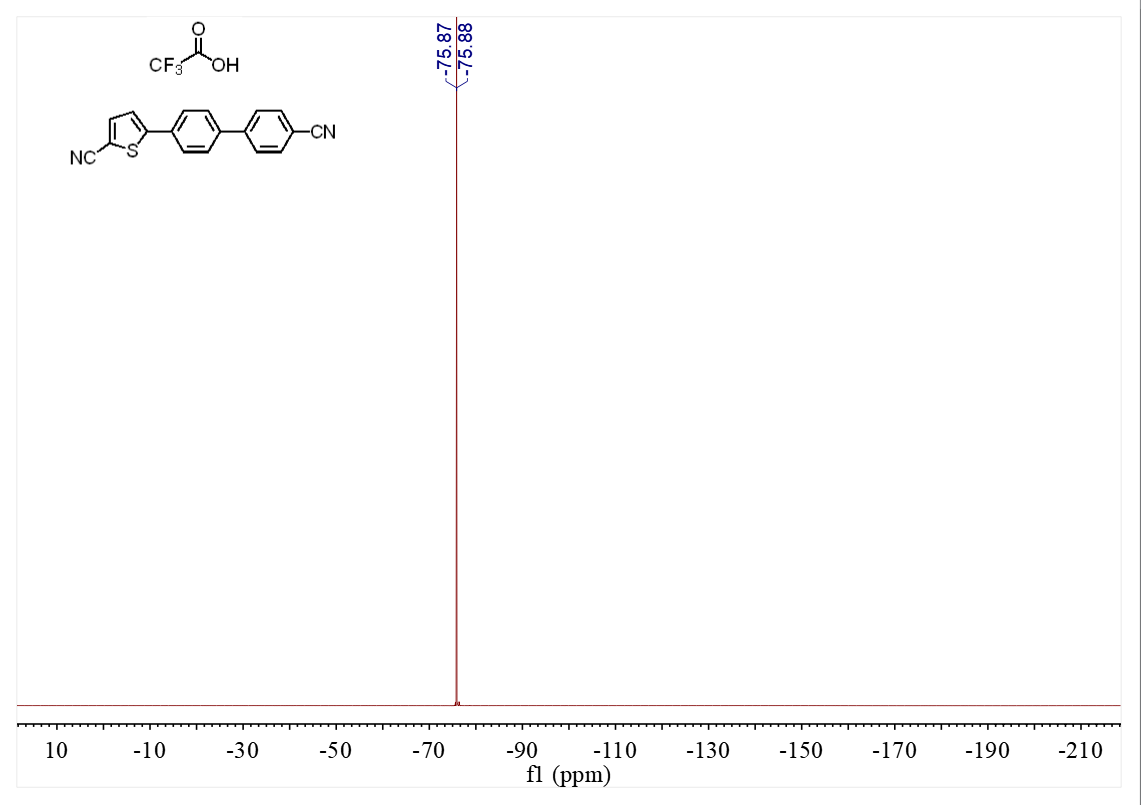


**Figure S11**. ^19^F NMR spectrum of CF_3_COOH.


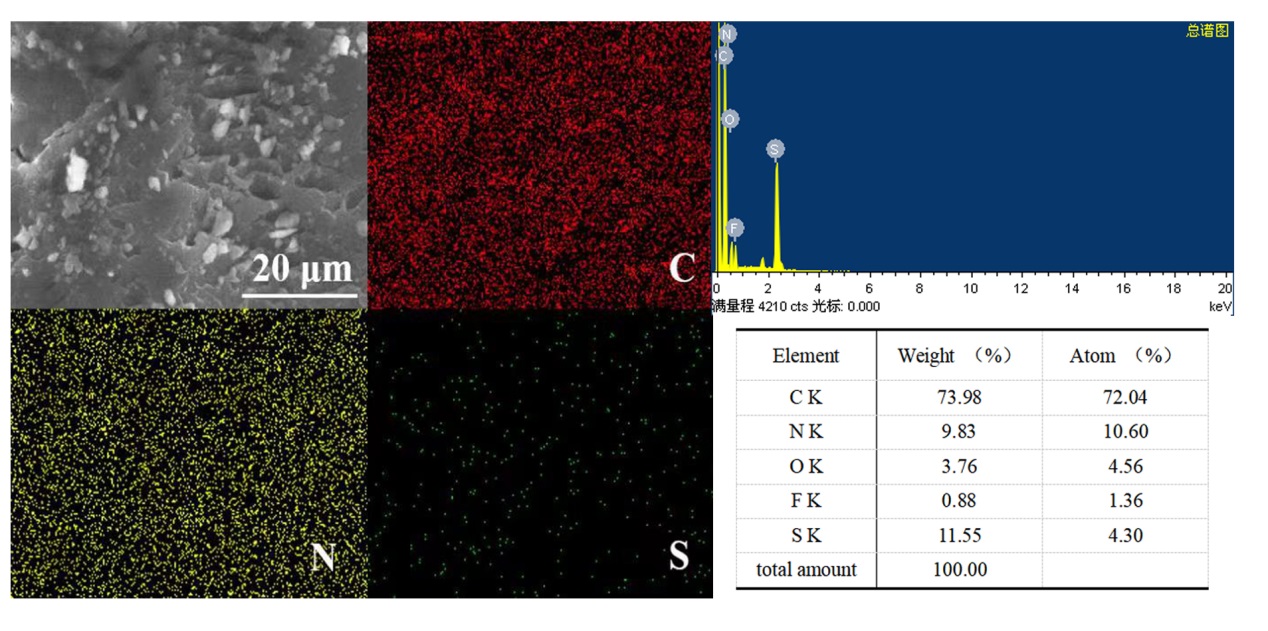


**Figure S12.** EDX elemental mapping and analysis of As-CTF-S.
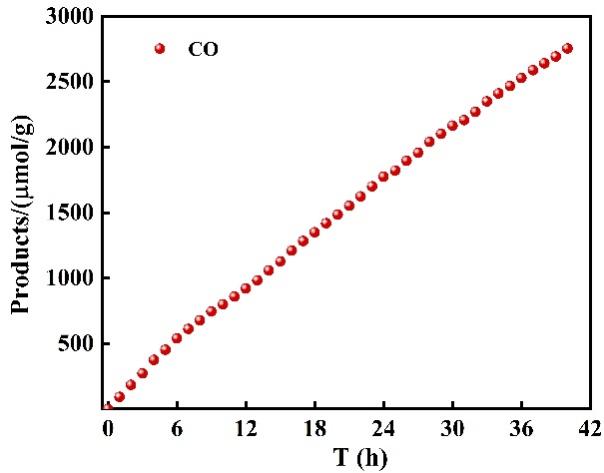


**Figure S13.** Time-course plots of products with As-CTF-S as the photocatalyst.

Table S1. The reported CTF-based photocatalysts for the CO_2_ photoreduction.

| Polymer | Sacrificial agent | Photoinitiator | Products | Yield  （μ mol /h/g） | References |
| --- | --- | --- | --- | --- | --- |
| Nonmetallic CTFs | |  |  |  |  |
| NCTF-1 | TEA |  | CH_4_ | 11.48 | [1] |
| SnS_2_/S-CTFs | TEOA |  | CO | 1200 | [2] |
| Metallized CTFs | | | | | |
| Re-CTF-py | CH_3_CN/TEOA |  | CO | 353.05 | [3] |
| Co/CTF-1 | CH_3_CN/TEOA/ H_2_O | [Ru(bpy)_3_Cl_2_]6·H_2_O | CO | 50 | [4] |
| CTF-TDPN | CH_3_CN/TEOA/ H_2_O |  | [CO:H_2_ = 1.4:1] | 330.3 | [5] |
| Fe SAS/Tr-COF | CH_3_CN/TEOA/ H_2_O | [Ru(bpy) _3_Cl_2_]·6 H_2_O | CO | 980.3 | [7] |
| Ru-CTF | DMA/TEOA |  | HCOOH | 2090 | [8] |
| Cu_0.1_ SA/CTF | TEA/ H_2_O |  | CH_4_ | 32.56 | [9] |
| Pd@Imine-CTF | TEOA |  | CO | 85.3 | [10] |
| 20%-Ni-CTAB-CTF-1 | CH_3_CN/TEOA/ H_2_O | [Ru(bpy) _3_Cl_2_]·6H_2_O | CO | 1254.15 | [11] |
| Ni (OH)_2_-CTF-1 | CH_3_CN/TEOA/ H_2_O | [Ru[bpy] _3_Cl_2_]·6H_2_O | CO | 38.66 | [13] |
| Pt-SA/CTF-1 | TEA/ H_2_O |  | CH_4_ | 4.6 | [14] |
| Photosensitized CTFs | | | | | |
| *α*-Fe_2_O_3_@Por-CTF | DMF/TEOA |  | CO | 8 | [12] |
| CTF-Bpy-Co | CH_3_CN/TEOA/ H_2_O |  | CO | 1200 | [6] |
| Donor-acceptor CTFs | | | | | |
| DA-CTF | CH_3_CN/TEOA |  | CO | 4 | [15] |
| CT-COF | H_2_O |  | CO | 102.7 | [16] |
| CTF-based hybrid systems | | | | | |
| CuCo_2_O_4_/CTF-1 | CH_3_CN/TEOA/ H_2_O | [Ru(bpy) _3_Cl_2_]·6 H_2_O | CO | 14.9 | [17] |
| CuSx/TP-TA | H_2_O |  | CO | 6.78 | [18] |
| CABB/CTF-1 | EA |  | CO | 122.9 | [19] |
| CsPbBr_3_/CTF-1 | EA |  | CO | 173 | [20] |
| TiO_2_@CTF-Py(CoCl_2_) | H_2_O |  | CO | 43.34 | [21] |
| NH_2_-MIL-68(In)@TPTA | H_2_O |  | CO | 25 | [22] |
| CTF-BP |  |  | CH_4_ | 7.81 | [23] |
| CN/CTF | CH_3_CN/TEOA |  | CO | 151.1 | [24] |
| As-CTF-S | H_2_O | No | CO | 88.3 | This work |

TDPN: 4,4′-biphenyldicarboxaldehyde; TEOA: triethanolamine; TEA: triethylamine; CH_3_CN: acetonitrile; bpy: 2,2′-bipyridine; DMA: dimethylacetamide; EA: ethyl acetate.

It’s clear from the description that two primary modes exist for photocatalytic carbon dioxide reduction: gas-solid and liquid-solid. In the liquid-solid mode, there’s a significant competing hydrogen evolution reaction. Commonly used organic solvents in this mode include acetonitrile and DMF, and sacrificial agents like TEOA are used associated with co-catalysts such as rhenium or cobalt pyridine complexes. This setup leads to a complex reaction system, increased costs due to gas separation, and potential secondary environmental pollution. In contrast, using water vapor as a sacrificial agent in the gas-solid mode appears more cost-effective and environmentally friendly. Among the catalysts for CO_2_ reduction, several CTF-based catalysts have been noted for their efficiency, operating without metals, co-catalysts, sacrificial agents, or photosensitizers. The As-CTF-S, in particular, achieves a high yield of 88.3 μmol/h/g and shows high product selectivity when water is used as the medium. However, the catalyst developed in our study outperforms these existing materials in both performance and selectivity, surpassing most similar types of catalysts currently reported in the literature.

References

[1] Q. Niu, Z. Cheng, Q. Chen, G. Huang, J. Lin, J. Bi, L. Wu, *ACS Sustainable Chem. Eng*. **2021**, *9*, 1333.

[2] S. Guo, P. Yang, Y. Zhao, X. Yu, Y. Wu, H. Zhang, B. Yu, B. Han, M.W. George, Z. Liu, *ChemSusChem.* **2020**, *13*, 6278.

[3] R. Xu, X.-S. Wang, H. Zhao, H. Lin, Y.-B. Huang, R. Cao, *Catal. Sci. Technol*. **2018**, *8*, 2224.

[4] J. Bi, B. Xu, L. Sun, H. Huang, S. Fang, L. Li, L. Wu, *ChemPlusChem*.**2019**, *84*, 1149.

[5] Y. He, X. Chen, C. Huang, L. Li, C. Yang, Y. Yu, *Chinese. J. Catal.* **2021**, *42*, 123.

[6] X. Hu, L. Zheng, S. Wang, X. Wang, B. Tan, *Chem. Commun*. **2022**, *58*, 8121.

[7] L. Ran, Z. Li, B. Ran, J. Cao, Y. Zhao, T. Shao, Y. Song, M. K. H. Leung, L. Sun, J. Hou, *J. Am. Chem. Soc*. **2022**, *144*, 17097.

[8] L. Wang, L. Wang, S. Yuan, L. Song, H. Ren, Y. Xu, M. He, Y. Zhang, H. Wang, Y. Huang, T. Wei, J. Zhang, Y. Himeda, Z. Fan, *Appl. Catal. B*. **2023**, *322*, 122097.

[9] G. Huang, Q. Niu, Y. He, J. Tian, M. Gao, C. Li, N. An, J. Bi, J. Zhang, *Nano Res*. **2022**, *15*, 8001.

[10] S. Guo, Y. Xiao, B. Jiang, *ACS Sustainable Chem. Eng.* **2021**, *9*,12646.

[11] J. Tian, J. Zhang, B. Xu, Q. Chen, G. Huang, J. Bi, *ChemSusChem.***2022**, *15*, 202201107.

[12] S. Zhang, S. Wang, L. Guo, H. Chen, B. Tan, S. Jin, *J. Mater. Chem. C*. **2020**, *8*, 192.

[13] T. Zhao, Q. Niu, G. Huang, Q. Chen, Y. Gao, J. Bi, L. Wu, J. *Colloid. Interface. Sci.* **2021**, *602*, 23.

[14] G. Huang, Q. Niu, J. Zhang, H. Huang, Q. Chen, J. Bi, L. Wu, *Chem. Eng. J.* **2022**, *427*, 131018.

[15] H. Zhong, Z. Hong, C. Yang, L. Li, Y. Xu, X. Wang, R. Wang, *ChemSusChem* **2019**, *12*, 4493.

[16] K. Lei, D. Wang, L. Ye, M. Kou, Y. Deng, Z. Ma, L. Wang, Y. Kong, *ChemSusChem*. **2020**, *13*, 1725.

[17] G. Lin, L. Sun, G. Huang, Q. Chen, S. Fang, J. Bi, L. Wu, *Sustain. Energy Fuels*. **2021**, *5*, 732.

[18] J. Mao, L. Wang, S. Qu, Y. Zhang, J. Huang, H. She, Y. Bai, Q. Wang, *Inorg. Chem*. **2022**, *61*, 20064.

[19] Z. Zhang, Y. Jiang, Z. Dong, Y. Chu, J. Xu, *Inorg. Chem*. **2022**, *61*,16028.

[20] Q. Wang, J. Wang, J.-C. Wang, X. Hu, Y. Bai, X. Zhong, Z. Li, *ChemSusChem* **2021**, *14*, 1131.

[21] Z. Xu, Y. Cui, D. J. Young, J. Wang, H.-Y. Li, G.-Q. Bian, H.-X. Li, *J.CO_2_ Util*. **2021**, *49*, 101561.

[22] L. Wang, J. Mao, G. Huang, Y. Zhang, J. Huang, H. She, C. Liu, H. Liu, Q. Wang, *Chem. Eng. J*. **2022**, *446*, 137011.

[23] J. Li, P. Liu, H. Huang, Y. Li, Y. Tang, D. Mei, C. Zhong, *ACS Sustainable Chem. Eng*. **2020**, *8*, 5175.

[24] J. He, X. Wang, S. Jin, Z.-Q. Liu, M. Zhu, *Chinese J. Catal.* **2022**, *43*,1306.
